# Investigating Sexual Characteristics in Two Frog Species Under Exposure to River Water Polluted with Endocrine Disruptors

**DOI:** 10.3390/ani15233364

**Published:** 2025-11-21

**Authors:** Martyna Frątczak, Mikołaj Kaczmarski, Katarzyna Szkudelska, Abdallah Yussuf Ali Abdelmajeed, Łukasz Jankowiak, Tomasz Maliński, Łukasz Myczko, Monika Ostaszewska, Anna Przybylska-Balcerek, Beata Rozenblut-Kościsty, Joachim Siekiera, Kinga Stuper-Szablewska, Piotr Tryjanowski

**Affiliations:** 1 Department of Zoology, Poznan University of Life Sciences, Wojska Polskiego 71C, 60-625 Poznan, Poland; 2 Department of Animal Physiology, Biochemistry and Biostructure, Poznan University of Life Sciences, 60-637 Poznan, Poland; 3 Department of Ecology and Environmental Protection, Poznan University of Life Sciences, 60-637 Poznan, Poland; 4 Department of Ecology and Anthropology, Institute of Biology, University of Szczecin, 70-415 Szczecin, Poland; 5 Department of Botany and Forest Habitats, Faculty of Forestry and Wood Technology, Poznan University of Life Sciences, 60-625 Poznan, Poland; 6 Research and Development Laboratory, Chespa Sp. z o.o., 47-316 Chorula, Poland; 7 Department of Chemistry, Poznan University of Life Sciences, 60-625 Poznan, Poland; 8 Department of Evolutionary Biology and Conservation of Vertebrates, University of Wrocław, 50-335 Wroclaw, Poland

**Keywords:** amphibia, digit ratio, endocrine-disrupting compounds, *Rana temporaria*, *Rana arvalis*

## Abstract

Endocrine-disrupting compounds (EDCs) are emerging environmental pollutants that are known to the disrsupt hormonal system of many vertebrates. Amphibians, with their aquatic larval stages and high sensitivity to waterborne contaminants, are especially vulnerable to EDC exposure. Despite increasing concerns over EDC pollution, systematic monitoring of these compounds in surface waters remains limited in many regions, including the European Union. This study investigates the effects of water from the Warta River, one of the largest rivers in Central Europe, an urban waterway subjected to significant anthropogenic pressure and known to contain EDCs on body condition, digit ratio, and gonadal development in two brown frog species: the common frog *Rana temporaria* and the moor frog *Rana arvalis*. We propose DR as a potential biomarker of endocrine disruption, as it is linked to hormonal impact in the early development of vertebrates. In this study, tadpoles were reared in the semi-open experimental setup with tanks containing river or potable tap water as a control. Contrary to expectations, no significant differences were observed in body condition, digit ratio, or gonadal structure, suggesting that EDC concentrations in the river water may not have been high enough to induce detectable effects. However, a consistent relation between DR and sex was observed in both species, underscoring its potential as a biologically meaningful trait. Notably, the potable tap water used as a control exhibited contamination levels comparable to the river water, raising concerns about the efficacy of current water treatment methods and highlighting the challenges of establishing true reference conditions in environmental studies.

## Simple Summary

Amphibians are considered excellent bioindicators of environmental pollution because of their high sensitivity to waterborne contaminants. One group of pollutants that has attracted increasing attention are endocrine-disrupting compounds—substances capable of interfering with hormonal regulation. Although such chemicals are present in many water bodies, routine monitoring is still limited. In this study, we explored whether water from the Warta River in Central Europe, which flows through urbanized areas and is subject to heavy human impact, influences the development of two frog species: the common frog and the moor frog. We also proposed digit ratio, the relative length of fingers, as a simple bioindicator of hormonal disruption. Tadpoles were raised in either river water or tap water and examined for body condition, sexual development, and digit ratio. We found no clear differences between groups, suggesting that pollutant levels were too low to cause visible changes. However, digit ratio was consistently linked to sex, confirming its biological relevance. Importantly, tap water contained contaminants similar to river water, raising concerns about drinking water treatment and showing how difficult it is to define truly clean reference conditions in ecological studies.

## 1. Introduction

Endocrine-disrupting compounds (EDCs) are exogenous substances, often of anthropogenic origin, that mimic or interfere with the action of naturally occurring hormones, leading to adverse effects on organisms [1]. These compounds can profoundly impact animal development, particularly by disrupting reproductive and thyroid system functions. EDCs from sources such as plastic waste, personal care products, and pharmaceuticals are now recognized as major environmental pollutants, to which both humans and wildlife are persistently exposed [1,2,3]. In aquatic environments, particularly in surface waters across Europe and globally, EDC pollution is alarmingly high [4,5,6,7,8]. However, in many regions, including Poland, there is no mandated monitoring of most EDCs in environmental, effluent, or tap water, leaving significant gaps in understanding their prevalence and effects (data from the Polish Great Voivodeship’s Regional Inspectorate for Environmental Protection, unpublished).

Numerous studies have demonstrated the adverse effects of EDCs on aquatic organisms, most extensively documented in fish [9,10,11]. However, amphibians are considered even more sensitive to EDCs, especially in the context of unique bi-phasic life history. Their aquatic embryonic and larval stages have limited physiological protection against waterborne contaminants. EDCs can easily penetrate their body through permeable egg envelopes, gills, and the unprotected, thin and highly vascularized skin. Additionally, hormone-dependent developmental changes during these life stages increase susceptibility to adverse effects of EDCs [12,13,14]. EDCs are suspected to contribute significantly to the global decline in amphibian populations [15,16,17,18,19].

Due to their sensitivity to EDCs, amphibians have been proposed as effective bioindicators for detecting these compounds in the environment [13,20]. Most studies, however, focus on individual EDCs tested at high, environmentally unrealistic concentrations under laboratory conditions, often using a single model species such as the African clawed frog *Xenopus laevis* [21,22]. There is a pressing need for studies investigating the effects of multiple EDCs, both individually and in combination, at environmentally relevant concentrations in natural settings. Expanding research to encompass different amphibian species exposed to EDCs in their native habitats could yield more comprehensive insights.

In our study, we focus on digit ratio (DR, or 2D:4D ratio) as a potential phenotypic marker for assessing the effects of EDCs on amphibians. Most often, DR reflects the relative lengths of the second and fourth digits (2D:4D) and is a sex-related trait in many species, including amphibians and other groups of vertebrates [23,24,25,26]. It has been linked to levels of steroidal sex hormones during early development and is sensitive to endocrine disruption [27,28]. In many species of vertebrates, DR exhibits sexual dimorphism, although the direction of this bias varies among taxa [29,30,31]. In most mammals [32] and tailed amphibians [23] females tend to show higher 2D:4D values, whereas in birds and reptiles, the opposite pattern has been reported [33,34].

Among anuran amphibians, data on DR are still limited, with the trait described in only several species, with highly variable results [26,31,35,36,37,38,39,40]. A male-biased ratio has been reported, for instance, in the hind limbs of *Leptodactylus podicipinus* [31] and across all limbs of *Pelobates fuscus* [40], whereas a female-biased pattern was observed in the forelimbs of *Engystomops pustulosus* [38]. Some other species, however, showed no sex-related differences in DR [31,36,38]. These inconsistences may reflect interspecific differences in hormone sensitivity or developmental patterns, as well as methodological limitations, including incorrect digit numbering (for more details, see Kaczmarski et al., 2021 [26]). The limitation in most studies could also be lack of allometric adjustment of DR, which may be influenced by age and individual growth rate of animals [40]. For the same reason, studying individual animals of the same developmental stage or age group is essential for reliable comparisons [26,40].

Nonetheless, current evidence suggests that DR is hormonally modulated and could potentially be useful as a non-invasive indicator of endocrine disruption, as beyond sex-related differences, DR may also change in response to external endocrine factors. Experimental studies have confirmed that DR patterns in amphibians are hormonally dependent and can be modified by exposure to androgens during metamorphosis [31]. Similar effects were reported in mammals, where exposure to the estrogenic compound bisphenol A altered DR patterns in rats, with changes persisting across generations [30]. The authors concluded that DR in adult rats may serve as a biomarker of prenatal exposure to low doses of endocrine disruptors. Considering the high sensitivity of amphibians to such compounds during early development, DR may play an even more important role as an indicator of EDC effects within this group of vertebrates.

To our best knowledge, no studies yet have comprehensively tested whether DR in amphibians could serve as a reliable indicator of EDC pollution in aquatic environments. Therefore, this study aimed to evaluate the impact of environmentally sourced water from a big Central European river called Warta—below an urban section of the river flowing through the city of Poznań, Poland, known to contain EDC pollution [6]—on the body condition, DR, and sex of two species of brown frogs: the common frog *Rana temporaria* and the moor frog *Rana arvalis*. These two species, while closely related, exhibit differences in genetic makeup, phenotypic characteristics, and ecological niches [41] potentially influencing their sensitivity to EDC pollution.

During our study, it was unexpectedly discovered that the potable tap water used as the control was contaminated to a similar degree as the river water. This revelation presented a challenge as it compromised the integrity of our control group. Despite meticulous planning and efforts to select a clean water source for the control, the presence of contaminants in both water sources revealed the complexity of environmental conditions and the difficulty in securing an ideal control group. Given the lack of readily available alternatives and the already ongoing exposition of animals, we proceeded with the study to gather as much valuable data as possible. It is important to acknowledge the limitations of our study due to the absence of a proper control group, and we emphasize the necessity for future research to address these challenges. Transparency regarding methodological issues is essential for the accurate interpretation of our results and to ensure the integrity of scientific inquiry. In the discussion section, we reflect on the implications of this limitation and underscore the need for cautious interpretation of our findings in light of this constraint.

## 2. Materials and Methods

### 2.1. Selected Species

The species examined in this study, *R. temporaria* and *R. arvalis*, belong to the group of brown frogs, which primarily lead a terrestrial lifestyle, with an aquatic phase generally limited to breeding and larval development [42,43]. This means that their exposure to endocrine-disrupting compounds (EDCs) in surface waters is limited to a relatively short, but crucial period. However, the two species differ in their choice of breeding habitats, which could influence their sensitivity to these contaminants [41,44].

*R. temporaria* is more flexible in its breeding site selection, reproducing in various water bodies, including small reservoirs, temporary puddles, fish ponds, and even large rivers when no other options are available [41,44]. Additionally, after reaching two years of age, this species hibernates in water, further extending its aquatic exposure [45]. In contrast, *R. arvalis* is more selective in its breeding preferences, favoring small water reservoirs and wetlands [41,44].

Unlike *R. temporaria*, *R. arvalis* hibernates exclusively on land [46], which reduces its contact with aquatic environments outside the breeding season. These ecological differences could potentially play a role in the species’ susceptibility to EDCs. Despite these breeding place variations, both species can be commonly found in oxbow lakes [47,48], which are seasonally filled with water directly from rivers. The presence of these species in such habitats justifies their selection for this study, in which we used water sourced from a river, with a goal to closely mimic a natural exposure scenario.

### 2.2. Study Permissions

The studied species, the common frog *Rana temporaria* and the moor frog *Rana arvalis*, are classified as “Least Concern” at the European Union level [49]. At the national level *R. temporaria* is under partial protection status, while *R. arvalis*, due to the decreasing populations in some regions in Poland, has a status of a strictly protected species [50]. The use of *R. temporaria* and *R. arvalis* in this study was approved by the Regional Directorate for Environmental Protection (Decision no. WS.6401.105.2023.MK.2, 24 March 2023) and the General Directorate for Environmental Protection (Decision no. DZP-WG.6401.193.2025.TŁ, 3. July 2023).

### 2.3. Experimental Setup and Rearing of Tadpoles

At the beginning of April, spawn with embryos of *R. temporaria* and *R. arvalis* were acquired from the environment, from 6 different localizations in Poznań and its vicinity, to avoid close kinship of specimens. Localizations were chosen based on the experience of our team in research of these species. Embryos were reared in control water until reaching the stage of free-swimming tadpole (25 Gosner stage; [51]), at which process of sexual differentiation of gonads starts [52]. After this point, tadpoles of the two species were randomly selected and divided into experimental and control tanks (each replicated four times). The physical placement of the tanks was randomized using an AI tool (OpenAI, GPT-4, San Francisco, CA, USA; March 2023), which generated a random sequence of tank positions to avoid location-related biases such as differences in light and temperature.

We used 240 tadpoles of each species (common frog and moor frog), divided equally between control and experimental groups (120 per group). We maintained tadpoles in single-species groups of 30 per tank (10 from each of 3 localities per tank). This number was chosen to ensure adequate replication and sufficient statistical power, while balancing ethical considerations and logistical constraints, such as possible mortality of tadpoles.

Tadpoles were reared until the moment of emergence of all 4 limbs and beginning of the tail atrophy, corresponding to the Gosner stage 42/43 [51] in the semi-open experimental setup, in tanks located outside, and therefore, in conditions close to the natural ones (such as natural temperature variance and natural light cycle) [53]. The recommended minimum water volume per tadpole is 3.7 L [54]. However, in our study, tadpoles were housed in groups of 30 per tank, each containing 150 L of water, ensuring optimal space and water quality. Tanks were made from HDPE (high density polyethylene), approved for contact with food. Tadpoles were fed with dried and boiled nettle leaves which were replenished once a week after eating the previous batch, so access to food was ad libidum. Apart from that, the larvae used naturally occurring feed sources, such as algae, bacteria, and sediment that accumulated in the experimental tanks, as well as small invertebrates. The welfare and condition of tadpoles were monitored every week.

### 2.4. Acquiring and Testing Water for Control and Experimental Groups

As the water source for the experimental group, we selected the Warta River, a major Central European river. Originating in central Poland, the Warta meanders through the Polish Plain in a northwesterly direction before joining the Odra River. With a total length of approximately 808.2 km, it is the second-longest river within Poland, and flows through the urban area of Poznań [55].

The water collection point (52.65858, 16.76575) was selected to represent the river’s flow and its sewage load. This site receives treated municipal and industrial effluents and is located approximately 35 km downstream from the largest sewage treatment plant in the area, situated in the city of Poznań. The location also allowed for direct water sampling from the river. Water from the river was collected using professional pumps (Hunter 2850) approved for contact with drinkable water, and tanks from HDPE approved for contact with food. Every week, 50% of the water in each tank was replaced with freshly delivered water—controlled, dechlorinated, drinkable tap water from Dębina intake [56] or water directly from the Warta River.

Samples of water for the analysis were taken every other week from 8 randomly chosen water tanks, after replacing water, starting from the first week of exposition. We aimed for controlling the concentration of 8 EDCs, known for impacting development of amphibians or suspected of such action. Description and summary of chosen compounds can be found in Table 1. Water samples were collected in pre-cleaned glass bottles. Immediately after sample collection, the bottle was kept on ice in a cooling box (temperature < 4 °C) and delivered within 24 h to the laboratory, where it was analyzed within next 24 h. Bottles containing the samples were coded to ensure that the laboratory analysis was conducted blindly. To analyze steroid hormones (estrone, 17β estradiol, ethinylestradiol, progesterone), methylparaben, propylparaben, nonylphenol, and bisphenol A in water samples, the dispersive liquid–liquid microextraction (DLLME) method was used. Dichloromethane was used as the extraction solvent, and methanol was the dispersion solvent. Intensive mixing using a Vortex mixer was used to increase extraction efficiency. The obtained extract was quantitatively analyzed using gas chromatography–mass spectrometry (GC-MS/MS). Standards of methylparaben, propylparaben, 4-nonylphenol, BPA, steroid hormones (estrone, estriadol, ethinylestradiol, progesterone), and extraction solvents had >99% purity (Merck, Darmstadt, Germany).

Additionally, to calculate food base availability in the water tanks, in the first week of exposition, a collective sample of water from all experimental and control tanks was taken for the analysis of chlorophyll A and phaeophytin A content. The analysis was repeated in the last week of exposition, this time by taking samples of water from randomly chosen experimental and control tanks. Analysis of concentration of chlorophyll A and phaeophytin A was performed by concentrating the seston on a glass fiber (Whatman GF/C filter), extracting of assimilation dyes with acetone and then measuring of absorption of light, at a specific wavelength, before and after acidification with hydrochloric acid.

### 2.5. Finalizing Exposition

The exposition of animals started in the last week of April 2023 and lasted 10 weeks, until individuals reached 43–45 Gosner stage. Then froglets were then placed in 8 terrestrial semi-open enclosures: two enclosures for individual frogs from the control group and two enclosures for individual frogs from the experimental group for each species (for rearing details see [88]), for the period of two months, to allow the completion of gonadal development [52]. During this period, froglets were fed with wild invertebrates that have access to the enclosures as well as larvae of crickets and cockroaches two times a week.

### 2.6. Measurements

At the beginning of September, animals were immobilized by anesthesia with the methodology according to Green, 2009 [89] with the use of tricaine mesylate (MS-222; Merck, Darmstadt, Germany), a derivative of benzocaine, given parenterally. Anesthesia was performed by total immersion of frogs in 500 mL of 0.5% MS-222 (about 500 mg MS-222/100 mL of decanted water) in a glass container. Frogs that were completely induced into anesthesia were assessed based on lack of movement and reaction to touch. After anesthesia, animals were weighed, measured, and photographed. After that, animals were euthanized by cutting the spinal cord and dissected.

Two characteristics were measured using a manual caliper (accuracy: 0.01 mm): SVL (snout–vent length, which, in anurans, corresponds to the total body length) and head width (HW). To minimize additional human-induced systematic error (observer effect), all specimens were measured by only one researcher (MF). To calculate the intraobserver error, digit measurements were carried out twice for 30 randomly selected frogs.

Each frog was photographed. Two photographs of each of the four limbs were taken, in accordance with the methodology adopted by the previous research [23]. Photographs were taken with a Pentax Optio WG-5 digital camera (Ricoh Imaging Company Ltd., Tokyo, Japan). These cameras were equipped with a feature shooting microscope with an LED backlit ring placed directly on the lens. The camera was positioned on a glass plate with a scale marker (PEAK S-1983-S; Peak Optics, Tokyo, Japan) which was applied to each subsequent limb, which enabled the maintenance of a standard distance from the camera to the fixed object. While taking photographs, constant light conditions were maintained.

### 2.7. Digital Measurements

Images of limbs were used for further analysis. Individual digits on limbs were numbered in accordance with the pattern presented by Kaczmarski et al., 2021 [26]. The computerized measurements of DR, using landmarks, were performed in accordance with the methodology described by Kaczmarski et al., 2015 [23]. Tmorph Gen 6 software (Sheets, version Gen 6, USA, 2000) as used to measure the distance between points (landmarks) corresponding to the length of the surveyed digits. This procedure was repeated for each limb of each frog twice. The order of the limbs analyzed was randomized.

### 2.8. Anatomical and Histological Analysis

Sex determination of all frogs was performed during the dissection of specimens in accordance with the methods described by Haczkiewicz and Ogielska, 2013 [90]. After opening the body cavity, morphology of gonads was examined using a stereo microscope. Based on gross gonadal anatomy, phenotypic sexes were identified and the presence or absence of morphological abnormalities was recorded. Then, the gonads were collected and fixed in natural anatomical positions in Bouin’s solution (Merck, Darmstadt, Germany) for 4 h, then rinsed several rounds in 70% EtOH until the solution was no longer yellowish, and finally stored in EtOH (70%) until further processing.

Histological preparations were made from gonads collected from 40 randomly chosen frogs (10 males and 10 females from experimental group, 10 males and 10 females from the control group) of the *R. arvalis* and 40 (10 males and 10 females from experimental group, 10 males and 10 females from the control group) of the *R. temporaria*. For histology sections, gonads were separated from adjoining tissues and embedded in Paraplast using standard procedures, sectioned on Leica RM 2255 microtome (Leica Biosystems, Nussloch, Germany) into 7 μm thick longitudinal sections, stained with Mallory’s trichrome, and examined using a Zeiss Axioskop 20 microscope. Images were acquired by a cooled Carl Zeiss Axio-Cam HRc CCD camera and ZEN blue imaging system (Carl Zeiss Microscopy GmbH, Oberkochen, Germany). Stages of gonadal development were assessed according to Haczkiewicz & Ogielska, 2013 [90]. Histological samples were screened slide by slide for confirmation of phenotypic sex identification, information on stage of gonadal development, and presence of histological alterations.

## 3. Data Analysis

### 3.1. Water Parameters

Weekly measurements of temperature, pH, and conductivity were analyzed to assess differences between groups and among tanks within each group. Since the data deviated from a normal distribution, pairwise Wilcoxon tests were used for comparisons. Statistical significance was set at *p* < 0.05.

### 3.2. Measurement Precision

For each digit, two independent measurements were taken. For the length of 2D (second digit), differences ranged from 0.00 to 0.61, with a mean absolute deviation of 0.065. For 4D (fourth digit) the differences ranged from 0.00 to 0.60, with a mean absolute deviation of 0.077. While most discrepancies were minor, occasional larger variations suggest potential measurement. To minimize the impact of these discrepancies, the mean values were used to further the analysis. The measurements of HW and SVL were repeated for 30 random frogs to calculate intraobserver error. For the HW, the absolute differences ranged from 0.03 to 0.48, with a mean discrepancy of 0.19 and a standard deviation of 0.15, while for SVL the absolute differences ranged from 0.00 to 0.34, with a mean discrepancy of 0.11 and a standard deviation of 0.09. Mean values of sexual differences in weight, SVL, and HW can be found in Tables S1 and S2, and of DR in Table S3.

### 3.3. Mortality

Mortality rates were analyzed using chi-square tests, with post hoc 2 × 2 chi-square comparisons to assess differences between species and groups at two stages of rearing (10-week exposure in water tanks and 2 months in terrestrial enclosures). Statistical significance was set at *p* < 0.05.

### 3.4. Body Condition

To assess potential differences in body condition between groups, sexes, and species, BMI (Body Mass Index) was recorded, calculated as mass divided by SVL^2^. Normality of BMI distributions were tested using the Shapiro–Wilk test, and due to non-normal distributions, the Mann–Whitney U test was used for non-parametric comparisons between experimental and control groups. A linear regression model was applied to assess the effects of group, sex, and species on BMI, with standard model diagnostics ensuring statistical validity.

### 3.5. Allometric Adjustment of Digit Ratios

To control for differences in digit size, an allometric adjustment was applied, ensuring that observed variations in DR were not confounded by overall digit length. Outliers were identified and removed separately for each species before model fitting. Individuals with extreme values in DR or SVL that fell beyond 1.5 times the interquartile range (IQR) were excluded to ensure statistical robustness.

This methodology was according to our previous study [40]. The adjustment was performed using a subgroup-specific scaling approach. For each combination of species (*Rana arvalis* [RA], *Rana temporaria* [RT]), sex (male/female), and limb type (forelimb [FL/FR], hind limb [HL/HR]), we calculated the mean length of the fourth digit (4D) within the subgroup. The adjusted DR for each individual was then derived as follows:

$$DR\_adj=\frac{{D2}_{adj}}{{mean\left(D4\right)}_{subgroup}}$$

where mean(D4) represents is the mean fourth digit length within each species–sex–limb subgroup.

This method minimized biases introduced by digit length size heterogeneity, allowing comparisons of digit proportions independent of their overall growth patterns. By standardizing within biologically homogeneous subgroups, we isolated potential sex-related or environmental effects on DR.

### 3.6. Statistical Analysis of Digit Ratios

To evaluate the influence of experimental exposure, sex, limb type, and species on the adjusted digit ratio (DR_adj), we conducted stratified linear regression analyses separately for *R. arvalis* (RA) and *R. temporaria* (RT). For each species, we fitted heteroscedasticity-robust ordinary least squares (OLS) models using HC3 standard errors. Predictor variables included group (experimental vs. control), sex, snout–vent length (SVL), and limb type (forelimbs [FL, FR] vs. hind limbs [HL, HR]). Fitting separate models for each species was crucial given the imbalanced sample sizes and potential differences in growth patterns, thereby ensuring comparability of the DR across subgroups.

Before modeling, we computed Pearson’s correlation coefficients between the second (D2) and fourth (D4) digit lengths across limb and sex subgroups to verify the consistency of our DR measurements. Post hoc comparisons among forelimbs/hind limbs, sexes, and species were performed using Tukey’s HSD tests.

Multicollinearity was assessed using variance inflation factors (VIFs), with all predictors showing VIF values below 2.5, thereby confirming negligible collinearity. All statistical analyses were performed in Python (v3.12) using *pandas* for data manipulation, numpy for numerical computations, seaborn and matplotlib for data visualization, scipy for statistical tests, and statsmodels for regression modeling and diagnostics.

## 4. Results

### 4.1. Conditions in Water Tanks

Based on weekly measurements, no significant differences in temperature were observed between control and experimental tanks (Figures S1 and S2), suggesting that both groups experienced similar thermal conditions. The temperature gradually increased over time, following natural oscillations during this time of the season. However, pH was significantly higher in the experimental group compared to the control group (*p* < 0.01), while the control group had significantly higher conductivity than the experimental group (*p* < 0.001). Conductivity and pH levels remained relatively stable over time, with slight fluctuations which could be due to regular water exchanges. There were no significant differences between tanks within each group in any parameter (*p* > 0.05, Bonferroni-adjusted) (Figure S2). Parameters of water in both experimental (E) and control (C) tanks remained within the optimal range for tadpole rearing [54,89], ensuring suitable developmental conditions for the larvae.

### 4.2. Concentrations of Endocrine Disruptors in Control and River Water

During the experiment, we detected several endocrine-disrupting compounds (EDCs), including methylparaben, propylparaben, nonylphenol, bisphenol A, estrone, estradiol, ethinylestradiol, and progesterone (Table 2).

In the first week of exposure, the highest concentrations of EDCs were observed. Methylparaben and propylparaben were abundant in tap water controls, with concentrations reaching up to 2.32 ppb and 0.97 ppb, respectively. Propylparaben was also detected in river water samples at a relatively high concentration (up to 4.59 ppb); however, surprisingly, no traces of methylparaben were found in these samples. Estrone was detected at a high concentration (4.53 ppb) in one river sample, while in a control sample it was present in much lower amounts (0.16 ppb).

Ethinylestradiol was prominent in all control and river water samples, with concentrations as high as 5.55 ppb in river samples and 3.42 ppb in control samples. Nonylphenol was detected in one control tank at a concentration of 0.17 ppb. Bisphenol A was present in all tanks, although at uncertain concentrations below 0.095 ppb. Estradiol and progesterone were also found at uncertain levels below the detection limit, in some river samples.

By the third week of exposure, the concentrations of all EDCs had declined significantly. Traces of nonylphenol, estradiol, and progesterone were no longer detected. By the fifth week, all EDCs in both control and experimental groups were either undetectable or below the limit of quantification (LOQ). Notably, ethinylestradiol remained detectable and measurable in both control and experimental tanks, with no significant differences in concentrations between groups. By the seventh and ninth weeks, all analyzed compounds were consistently undetectable or below LOQ across both control and experimental water samples, indicating a complete or near-complete disappearance of EDCs.

### 4.3. Chlorophyl A and Pheophytin A Content

The experimental (E) tanks initially exhibited higher concentrations of both chlorophyll A and pheophytin A (Table 3), indicating more established algal assemblages. In comparison, the control tanks began with much lower chlorophyll A levels (around 1.28 µg/L during the first week) but experienced a more pronounced increase over time, reaching up to 152.36 µg/L by the ninth week.

### 4.4. Mortality

**Figure 1:**
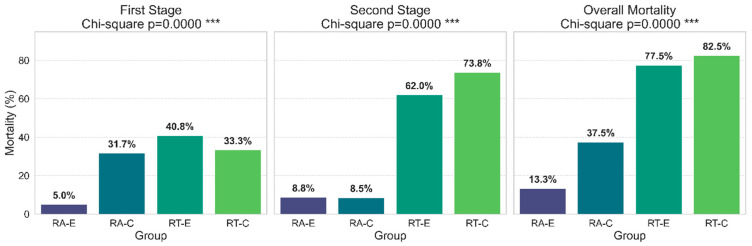
Mortality in *Rana arvalis* (RA) and *Rana temporaria* (RT), experimental (E) and control (C) groups, during first stage (10-week exposition in water tanks) and second stage (2 months in terrestrial enclosures) of rearing, as well as overall mortality for the whole study period. Differences were statistically significant (*** *p* = 0.0000).

Following a 10-week exposure period and a subsequent 2-month rearing phase in terrestrial enclosures, a number of individuals failed to survive (Figure 1). During the first stage of rearing (10-week exposure in water tanks), the experimental group (E1-4) of *R. arvalis* had significantly lower mortality than all other groups (*p* < 0.0001). No significant differences were observed among the control group of *R. arvalis*, and both experimental and control groups of *R. temporaria*.

In the second stage of rearing (two months in terrestrial enclosures), mortality was similarly low in both control and experimental groups of *R. arvalis* (~8.6–8.8%). However, mortality in both groups of *R. arvalis* differed significantly from the control and the experimental group of *R. temporaria* (*p* < 0.0001), which experienced high mortality (62.0–73.8%). No significant difference was found between the control and experimental group of *R. temporaria.*

The number of successfully metamorphosed individuals, which entered subsequent analyses, were as follows: 179 *R. arvalis* frogs (75 from the control group and 104 from the experimental group) and 48 *R. temporaria* frogs (21 from the control group and 27 from the experimental group).

### 4.5. Body Size and Condition

**Figure 2:**
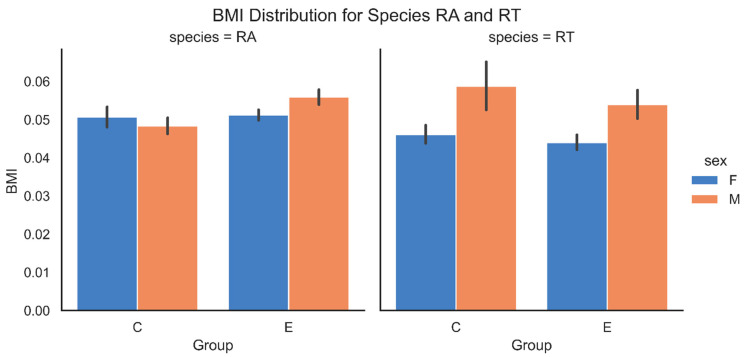
Body Mass Index (BMI) across control (C) and experimental (E) groups in *Rana arvalis* (RA) and *Rana temporaria* (RT).

Body size parameters varied between groups, sexes, and species, due to the relationship between weight and SVL differences (Figures S3 and S4). While neither group nor species alone had a significant effect on weight or SVL, a strong interaction between group, sex, and species was observed for both weight and SVL (*p* < 0.01). Males in the experimental group were significantly heavier and had longer SVL compared to the control group, particularly in *R. temporaria*. Body Mass Index (BMI) calculated based on weight and SVL in *R. arvalis* (Figure 2) remained comparable between groups, with some sex-based differences. In *R. temporaria*, males consistently had higher BMI than females, and the experimental group exhibited overall higher BMI values than the control one.

### 4.6. Deformities of Rana temporaria Digits

**Figure 3:**
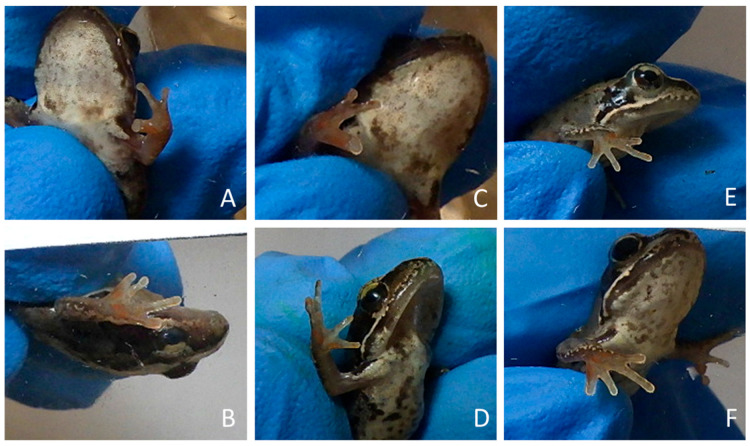
Syndactyly in forelimbs observed in 4 frogs of *Rana temporaria*. In 2 of them, abnormality was observed on both left and right sides (**A**–**D**), while in remaining 2 only on the right side (**E**,**F**).

In four frogs from the *R. temporaria* control group, we recorded developmental abnormalities: syndactyly in the digits of the forelimbs, where two digits appeared fused or not fully separated (Figure 3) [91]. In two of these frogs, the syndactyly was present in both the left and right forelimbs, while in the remaining two, the abnormalities were limited to the right forelimb. Deformed limbs of these frogs were excluded from the DR analysis.

### 4.7. Digit Ratio: Patterns and Correlations

**Figure 4:**
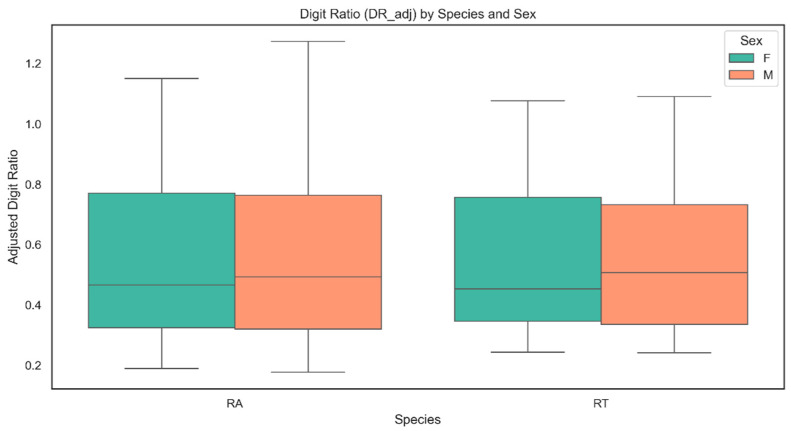
Differences in adjusted digit ratio between females (F) and males (M) in *Rana arvalis* (RA) and *Rana temporaria* (RT), without distinction between experimental and control groups. In both species, males exhibited significantly lower adjusted digit ratio (DR_adj) values than females (*p* < 0.001).

**Figure 5:**
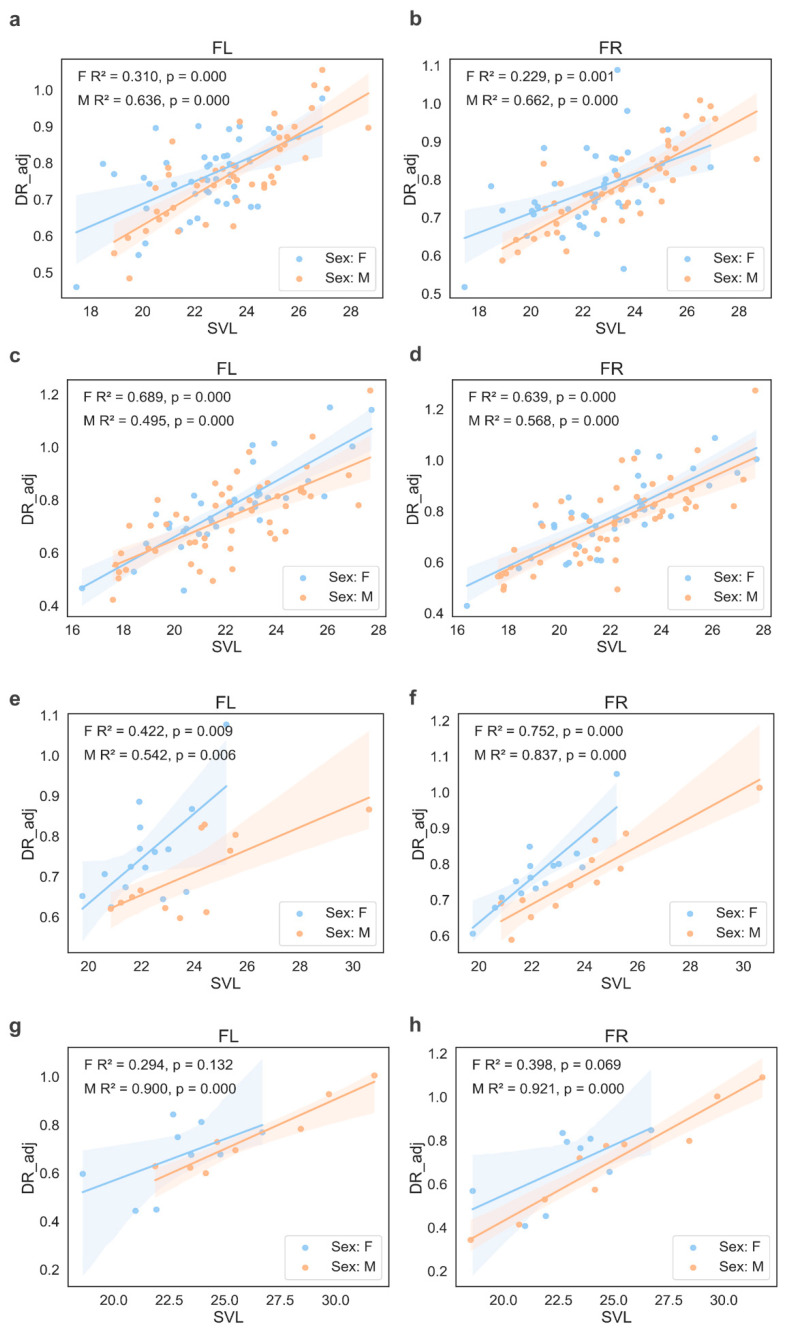
Relationship between snout–vent length (SVL) and adjusted digit ratio (DR_adj) in left (FL) and right (FR) forelimbs in females (F) and males (M) in (**a**,**b**): *Rana arvalis*, the experimental group; (**c**,**d**): *R. arvalis*, the control group; (**e**,**f**): *Rana temporaria*, the experimental group; (**g**,**h**): *R. temporaria*, the control group. A significant positive correlation was observed between SVL and DR_adj, indicating that larger individuals had higher 2D:4D digit ratios. This pattern was consistent across both species, in both control and experimental groups, and for both forelimbs and hind limbs.

The analysis revealed clear differences in digit ratio (DR_adj) between sexes, groups, and species (Figure 4). In *R. arvalis*, males had significantly lower DR_adj than females (*p* < 0.001), confirming a strong sex-based effect. Moreover, in *R. arvalis* individuals from experimental groups exhibited lower DR_adj values compared to the control one (*p* < 0.001). Body size, measured as snout–vent length (SVL), was positively associated with DR_adj, with larger individuals showing higher values (*p* < 0.001) (Figure 5 and Figure S5). Limb type was another significant predictor, with hind limbs (HL and HR) consistently linked to lower DR_adj values (*p* < 0.001). The model explained 90.6% of the variance (R^2^ = 0.906), indicating a strong fit.

In *R. temporaria*, males also had significantly lower DR_adj than females (*p* < 0.001), demonstrating a consistent sex-related difference. However, in contrast to the *R. arvalis*, frogs in the experimental group had significantly higher DR_adj values than those in the control group (*p* < 0.001). The relationship between SVL and DR_adj was again positive (Figure 4 and Figure S5), reinforcing the pattern that larger individuals exhibited higher DR_adj values (*p* < 0.001). As in *R. arvalis*, limb type remained an important factor, with hind limbs (HL and HR) associated with lower DR_adj (*p* < 0.001). The model explained 89.7% of the variance (R^2^ = 0.897).

### 4.8. Sex Identification and Histological Examination of Gonads

#### 4.8.1. Sex Ratio

The sex ratio remained close to the expected 50:50 distribution in both the control (43% F, 57% M) and experimental (49% F, 51% M) groups of *R. arvalis*, as well as the experimental group of *R. temporaria* (56% F, 44% M). In the control group in *R. temporaria,* a male-biased ratio was observed (37% F, 63% M).

#### 4.8.2. Histological Examination of Gonads

**Figure 6:**
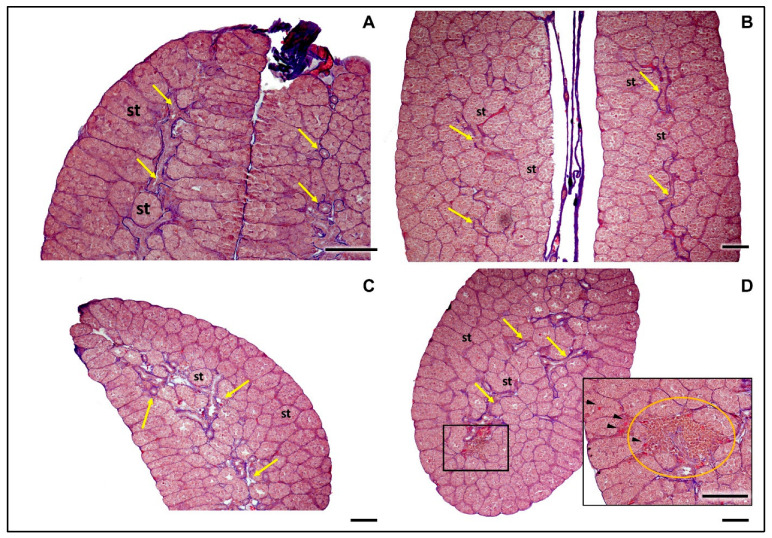
Transverse sections of morphologically normal Stage VIII testes of *Rana temporaria* from (**A**,**B**) control and (**C**,**D**) experimental groups. (**A**–**D**): Compact and well-organized seminiferous tubules (st) filled with gonocytes are observed. No secondary spermatogonia or meiotic cells are present, which is typical for this stage. The rete testis is well developed (yellow arrows). A notable characteristic is the absence of lumina within the seminiferous tubules. (**D**): In this gonad a small area of cellular degeneration can be seen, with an accumulation of orange-red somatic mesenchymal cells (orange circle). Surrounding this region, some degenerating gonocytes are still observable, indicating localized degeneration. The seminiferous tubules remain intact and physiologically normal, with only a few degenerating cells (black arrowheads). Overall, the testis demonstrates a healthy and expected developmental structure for this stage. Scale bar 100 µm.

The histological analysis of the testes of *R. temporaria* from both the control and experimental groups in our study revealed a consistent, morphologically normal appearance (Figure 6).

According to Ogielska, 2009 [52] in *R. temporaria* and other anurans testis develop in ten stages, starting as an undifferentiated gonad (Stage I) and maturing into a fully developed testis (Stage X). Testes found in our study were in stage VII-VIII of development [90], characterized by the presence of compact and well-organized seminiferous tubules filled with gonocytes (male germ cells) [92]. No secondary spermatogonia or meiocytes were observed at this stage, which aligns with this developmental phase. A defining feature was the absence of distinct lumina within the seminiferous tubules. In most of the frogs, the rete testis was already visible, further confirming the stage of maturation. The overall structure of the seminiferous tubules and surrounding tissue appeared healthy and normal, with no signs of degeneration or abnormalities.

**Figure 7:**
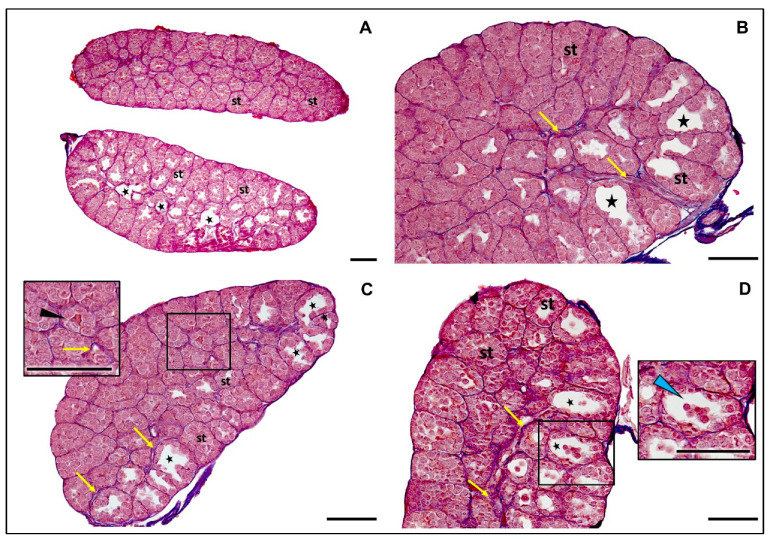
Transverse sections of overall, morphologically normal testes of *Rana arvalis* from (**A**–**C**) control and (**D**) experimental groups. (**A**): Stage VI testes. No secondary spermatogonia or meiotic cells are present. The seminiferous tubules (st) are visible and filled with gonocytes. The presence of a lumen within the tubules is observed in one testis, notably large in some of them (star marker). (**B**–**D**): Stage VIII testes. The rete testis is already developed (yellow arrows). The seminiferous tubules (st) are well-defined and filled with gonocytes. Some tubules exhibit a prominently large lumen, occasionally with gonocytes entering (star markers). Isolated degenerating cells are visible (black arrowhead). (**D**): The clear presence of numerous gonocytes entering the lumen of some tubules (blue arrowhead) in this testis suggests a degenerative process, possibly associated with cell removal. Scale bar 100 µm.

Similarly, testes of *R. arvalis* males in our study were found to have a well-defined structure characteristic of stage VIII of development (Figure 7).

The seminiferous tubules were prominent and filled predominantly with gonocytes. There was a noticeable absence of secondary spermatogonia and meiocytes, consistent with this developmental stage in related species [52,90]. However, an interesting feature was observed—in 7 out of 10 males in the control group and 8 out of 10 males in the experimental group, testes were notably large and lumina was observed within the seminiferous tubules (Figure 7). In the remaining males, 3 out of 10 in the control and 2 out of 10 in the experimental groups, the seminiferous tubules appeared tightly packed, exhibiting a dense and orderly structure, which could suggest slightly earlier, VII developmental stage.

**Figure 8:**
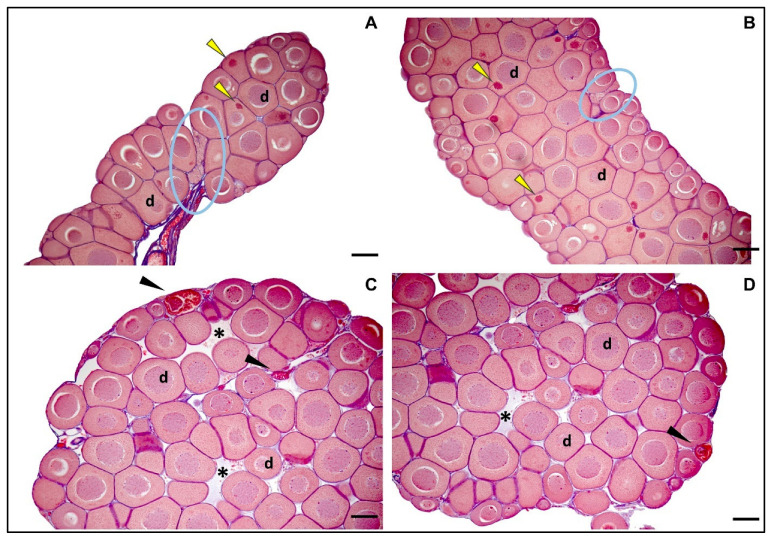
Transverse sections of physiological Stage IX ovaries. (**A**,**B**): *Rana temporaria*, female from the control group. The cortex is composed mainly of pre-vitellogenic diplotene oocytes (d), with well-defined Balbiani bodies (yellow arrowheads). Additionally, a germ patch, marked by a distinct cluster of cells (blue circle) is visible—this feature is typical for developing ovaries at this stage. (**C**,**D**): *Rana arvalis*, female from the control group. Transverse section of a physiological Stage IX ovary, showing a similar composition of pre-vitellogenic diplotene oocytes (d). Additionally, ovarian cavity (asterisk) is present. A few degenerating oocytes, a normal occurrence at this stage, are also visible (arrowheads). Scale bar 100 µm.

Physiological ovarian differentiation in *R. temporaria*, analogically to testes, can be divided into histologically characteristic stages, from stage I (undifferentiated gonad) to stage X (fully developed ovary) [93]. In our study, all females of *R. arvalis* and *R. temporaria* in both the control and experimental groups exhibited normal ovarian structure of IX stage of development (Figure 8). The ovaries contained properly developed diplotene oocytes, with only occasional degenerating cells observed, and with well-visible Balbani bodies, a mitochondria-rich structure, which is consistent with physiological processes at this stage [93]. In *R. temporaria*, ovarian Stage IX is typically reached about four weeks after metamorphosis [93] which is consistent with the Gosner stage 43–45 of frogs analyzed in our study.

## 5. Discussion

### 5.1. Conditions in Water Tanks

In the course of our study, when comparing the development of tadpoles in river water (experimental groups) versus tap water (control groups), it became evident that our intended control group exhibited roughly similar levels of contaminants (within the scope of the study) to that of the river water. In both river and tap water, propylparaben, nonylphenol, bisphenol A, estrone, estradiol, ethinylestradiol, and progesterone were detected (Table 2). In the tap water, additionally, methylparaben was found. This finding underscores not only the difficulty of selecting an appropriate control group in ecological studies but also raises concerns about the effectiveness of water treatment processes [94]. Despite being designated as potable, tap water still contained pollutants that could potentially influence biological responses.

In subsequent weeks of the experiment, we observed a decrease in EDC concentrations over time (see Table 2), which could be attributed to several factors. One key factor was that not the entire volume of water in the tanks was replaced each week. We limited it to 50%, to ensure the welfare of the tadpoles. The large-scale water replacements and sudden changes in water parameters could induce physiological stress, potentially leading to feeding disruptions, growth impairments, or increased mortality.

Additionally, the adsorption of EDCs onto organic matter accumulating at the bottom of the tanks, which mimicked natural conditions, likely contributed to their declining concentrations [95,96,97]. Seasonal fluctuations in river pollution, and consequently in tap water, may have also played a role in the observed decline. Previous research on EDCs and pharmaceuticals [98,99] has shown that pollutant concentrations in wastewater and drinking water treatment plants exhibit significant seasonal variation, with higher levels detected in winter than in summer. This is likely due to accelerated degradation rates at higher temperatures and greater sunlight exposure, as photodegradation [100,101] and microbial activity are key factors in reducing EDC levels [102,103]. Similarly, in our study, EDC concentrations were higher at the beginning, in April (first and third weeks of exposure), when ambient temperatures and UV light levels were relatively low. As the months progressed into May (fifth and seventh weeks) and June (ninth week) (Figure S1), rising temperatures and extended daylight hours likely enhanced the breakdown of EDCs in river water before sampling, which could explain the decreasing concentrations in the water tanks.

The randomized spatial arrangement of the water tanks may also account for variations in EDC concentrations among them. Although weekly measurements indicated no significant temperature differences between the tanks (Figure S2), slight variations in this parameter, along with differences in UV exposure, could have influenced the rate of EDCs degradation.

Notably, ethinylestradiol exhibited a slower degradation rate compared to estradiol and estrone. This characteristic likely contributed to its prolonged presence, explaining its persistence during the middle weeks of the study. As a synthetic analog of a natural hormone, its slower breakdown highlights its potential for greater and more prolonged harmful impacts on ecosystems [85]. This highlights the need for increased attention to its environmental persistence and potential ecological consequences.

The surprising presence of methylparaben in tap water but its absence in river water can be attributed to differences in sources. For example, river pollution may primarily stem from industrial effluents or agricultural runoff, which are less likely to contain methylparaben compared to personal care products that are more common in municipal wastewater [104], from which tap water may be sourced.

Moreover, the algal biomass could also play a role in the fate of EDCs in the water. Algae are known to contribute to the degradation of certain chemical compounds, either through direct uptake or by creating environmental conditions that promote chemical breakdown [103] (Im & Löffler 2016). Thus, tanks with higher chlorophyll levels may exhibit enhanced decomposition of EDCs, potentially reducing the concentration of these compounds over time, though this effect was not evident in our experiment.

Initially, experimental tanks with river water had higher chlorophyll A and pheophytin A concentrations (Table 3), indicating more established algal assemblages. In contrast, control tanks began with lower chlorophyll A levels but experienced a more pronounced increase over time, likely due to the semi-open setup and UV exposure, which facilitated algal growth. Some tanks showed less variation, possibly due to spatial differences affecting temperature and UV exposure.

The observed differences in chlorophyll A concentrations between the tanks could have another significant implication. Higher chlorophyll levels indicate greater algal biomass, which serves as a primary food source for tadpoles [105,106]. Variations in algal availability could, therefore, directly impact the tadpoles’ food supply, potentially influencing their growth rates, development, and overall condition [107,108].

### 5.2. Mortality and Condition of Individuals

Differences in food availability in the experimental tanks may explain the variation in mortality between groups at this stage of rearing (Figure 1). In *R. arvalis* the experimental group had significantly lower mortality compared to the control group. It suggests that better food availability—or other factors in the river water unmonitored by us—positively influenced early survival in this species. However, in *R. temporaria*, mortality remained similar between the control and experimental groups (31.7–40.8%), which could indicate that this species is more sensitive to other stressors.

After completing metamorphosis, during the 2-month rearing on land, *R. arvalis* in both groups had similar low mortality, while *R. temporaria* groups experienced very high mortality (approximately 62.0–73.8%). We hypothesize that this could be linked to genetic and developmental issues within the local population. Populations of *R. temporaria* in the study region are known to be in decline [109] (Pabijan & Ogielska 2019), likely due to environmental stressors (droughts, habitat degradation, and pollution) and genetic factors. The genetic issues could also explain the digit malformations observed in this group (Figure 3), which we discuss in detail below.

While some differences between species and groups in mass and condition were noted, the BMI values in surviving animals, across both groups, sexes, and species did not significantly differ from those previously recorded under optimal rearing conditions, based on our team’s experience with these species (unpublished data).

### 5.3. Syndactyly in Rana temporaria

In four individuals from the *R. temporaria* control group, we observed syndactyly in the forelimb digits (Figure 2). Although such anomalies are relatively common, they are often underreported. Due to their low frequency (<2.5%), they are generally considered within the range of natural variation, yet their underlying causes remain largely unknown. However, documenting these occurrences is crucial, particularly in assessing their prevalence within a population [91].

Notably, some studies have linked limb deformities to environmental pollution, highlighting the need for further investigation into potential external factors contributing to these anomalies. For example, similar abnormalities of digits, have been reported in *Bombina orientalis*. Populations of this species exposed to human activity and pollutants had increased incidence of such deformities [71]. Similarly, research conducted in Argentina has identified a higher prevalence of limb deformities in several amphibian species inhabiting pesticide-contaminated ponds, further supporting the role of environmental pollution in developmental anomalies [110]. Comparable digit malformations were also observed in *Hoplobatrachus tigerinus* in a study by Srabantika et al., 2014 [111], where three frogs found in the environment had only three digits in forelimbs and exhibited significant chromosomal abnormalities and one animal had mixed-sex gonads (possessing both testis and an oviduct). Authors suggested that these pathologies could be linked to environmental exposure to pesticides and other pollutant agents.

Notably, all cases of syndactyly occurred exclusively in the control group, suggesting that these anomalies were not induced by the direct exposure to EDCs. We also did not assess digit morphology during earlier developmental stages, so it remains unclear whether these deformities were present in a larger number of frogs that did not survive, including those in the experimental group. There is a need for future studies to evaluate the condition of *R. temporaria* populations in the region and investigate the prevalence of digit deformities in their natural habitats.

### 5.4. Digit Ratio

To our best knowledge, our study is the first to investigate DR in *R. arvalis* and *R. temporaria*, providing novel insights into the relationship between sex, body size (SVL), and DR variation in these species. Our results confirm strong and consistent effects of sex and body size on DR, with males exhibiting lower adjusted DR than females in both species, reinforcing a sex-related difference. Additionally, SVL was positively correlated with adjusted DR, indicating that larger individuals had higher digit ratios.

Data on DR in amphibians is highly limited. Although a number of studies on DR in this group of vertebrates was published, many of them used an incorrect scheme of numbering digits in forelimbs [26] and did not take into account the possible allometric effect of digits’ length [40] which makes them difficult to compare with our results.

The pattern of lower 2D:4D ratios in males compared to females observed in our study aligns with findings on *Bufo bufo*, where a similar trend was noted for non-adjusted 2D:4D in the right forelimb [40], while an opposite trend, with higher non-adjusted 2D:4D in males than females, was observed in hind limbs of *Oophaga pumilio* [35] and *Leptodactylus podicipinus* [31]. Also in our previous study [40] on the *P. fuscus*, in which we implemented allometric adjustment, a pattern of DR was different than the one observed in *R. arvalis* and *R. temporaria*. In *P. fuscus*, a higher adjusted 2D:4D ratio in all limbs was noted in males. Additionally, SVL was negatively associated with 2D:4D ratio.

The variation in DR patterns may stem from species-specific differences, as *P. fuscus* represents Mesobatrachia, which is a relatively primitive group of amphibians, while *R. arvalis* and *R. temporaria*, as well as *B. bufo* belong to Neobatrachia, which is a much more diverse and evolutionarily advanced group [42]. Notably, forelimb 2D and 4D in anurans play a key role in amplexus, as they support grasping behavior in males, with nuptial pads enhancing their function [112]. Potentially, variations in amplexus type across amphibians may contribute to sex differences in digit length and DR in forelimbs. While Mesobatrachia typically retain the ancestral inguinal amplexus, in which males grasp females near the hind legs, Neobatrachia predominantly use axillary amplexus [113,114]. As *R. arvalis* and *R. temporaria* are evolutionarily closely related [42] a similar pattern of DR and its relation to sex in these species is to be expected.

In our study we observed differences in allometrically adjusted DR between the experimental and control group; however, they were not consistent among species. In *R. arvalis*, frogs from the experimental group had lower DR_adj than the control group, whereas in *R. temporaria*, the opposite trend was observed. However, due to the non-model nature of the control group and differences and fluctuations in substances within the water tanks, drawing far-reaching conclusions for this finding should be avoided. The observed patterns highlight the interplay between morphological traits and external factors in shaping DR variation [26].

Notably, water from the Warta river reaches breeding sites of *R. arvalis* and *R. temporaria* [47]. Levels of EDCs in tanks observed in our study were probably within the range found in the natural environment of these species. This suggests that the observed sex-based differences in DR are likely to occur in natural populations, which reinforces the ecological relevance of our findings.

Overall, our study establishes a baseline understanding of DR variation in *R. arvalis* and *R. temporaria* and emphasizes the influence of morphological traits, species-specific factors, and environmental variability. Future research should incorporate controlled experimental conditions, larger sample sizes, and allometric adjustments to improve the accuracy of DR comparisons across amphibians.

### 5.5. Sex Ratio and Gonadal Development

In natural conditions, amphibians are exposed to many co-occurring compounds in water, which can have adverse effect on sexual development [115]. One study, that adopted an approach similar to our project, tested the impact of different concentrations (0%, 10%, 50%, 100%) of municipal wastewater effluent on the eggs and larvae of *Lithobates pipiens*. Wastewater, with a cocktail of EDCs, impaired gonadal development of males, causing occurrence of oocytes within testicular tissue [116]. In our study, we used water straight from the river, a water source reaching breeding sites of *R. temporaria* and *R. arvalis* in a region of the Greater Voivodeship in Poland, which, as we initially hypothesized, due to the number of EDCs present in it (Table 2), could negatively impact development of gonads and condition in these species.

The sex ratio in both species and groups, except for the *R. temporaria* control group, remained close to the expected 50:50 distribution. In the *R. temporaria* control group, a male-biased ratio was observed (37% F, 63% M); however, this was most likely a result of the small sample size due to high mortality, rather than an environmental influence.

The histological analysis revealed that all females of *R. arvalis* and *R. temporaria* in both the control and experimental groups exhibited normal ovarian structure, which was consistent with the somatic stage of animals. Similarly, males of *Rana temporaria* from both the control and experimental groups in our study had a consistent, morphologically normal appearance of gonads.

In *R. arvalis*, however, an unexpected feature was observed in majority of males in the control and experimental groups. In the testes of these males large lumina were found within the seminiferous tubules. This phenomenon may not specifically indicate a pathology, but represents a natural stage of maturation and suggests that development of testes in *R. arvalis* has a more accelerated rate compared to *R. temporaria*. In *R. temporaria*, seminiferous tubules remain compact until active spermatogenesis begins, after the first hibernation [52], leading to their significant expansion and the formation of larger lumina (corresponding to the developmental stage IX of the gonad). The presence of lumina in *R. arvalis* in testes before the first hibernation may indicate an earlier step toward maturity. This theory could be supported by the observations by Berger & Rybacki, 1993 [43], which reported that individuals of *R. arvalis* matured earlier than *R. temporaria*, and males already exhibited external sexual features before the first hibernation.

Alternatively, large lumina could suggest potential degeneration or the removal of cells into the tubule lumen. However, the degree of degeneration was relatively low, with degenerating cells sparsely distributed (see Figure 7). Interestingly, the large lumen appeared to usually affect only one gonad. It has been suggested that in Anurans, the left gonad is usually more advanced in development than the right one [52,117] which could confirm that observed changes are not a pathology.

Regarding morphological changes during normal testes development, the process is similar in *R. arvalis* and *R. temporaria* [117]. However, to the best of our knowledge, a detailed histological description of testis development in *R. arvalis* is not available in the existing literature, which makes it difficult to refer our findings to exemplary normal development of male gonads in this species.

Importantly, the large lumina observed in our study have not been documented in previous research. This includes studies on the physiological development of gonads in *R. temporaria* [90,118] and studies on the effects of endocrine-disrupting chemicals on the gonads of *R. temporaria* [77,119,120] and related species [82,121,122,123,124,125,126]. These findings suggest that expansively dilated lumina at this somatic stage of development, as identified in our observations, may represent a previously unexplored phenomenon.

Notably, in *Rana arvalis* and *Rana temporaria*, gonads originate from a bipotential primordium that differentiates into testes or ovaries under hormonal and genetic control [52,93]. In the species of brown frogs, the process of gonadal differentiation only begins during the aquatic larval phase and further continues after metamorphosis, when juveniles leave the water environment [82,93]. Consequently, although exposure to EDCs coincides with hormonally sensitive early stages, the continuation of gonadal development in terrestrial conditions may reduce the direct impact of waterborne endocrine disruptors compared to species completing gonadal differentiation entirely in the aquatic environment, such as *Pelophylax* kl. *lessonae* [93].

## 6. Conclusions

In our experiment, we tested the influence of water from the Warta River, one of the biggest river in Central Europe, below urban area, containing several EDCs, on the development of *R. arvalis* and *R. temporaria*. Our study showed that water sourced from Warta, despite presence of some EDCs, did not significantly affect the sexual development and gonadal structure of these species, which is optimistic from a conservational point of view. However, more data on the physiological development of male gonads of *R. arvalis* as a point of reference is needed to better interpret the results. We also did not observe a negative effect of exposure to river water on body condition of animals.

As the key result of the study, we consider consistent effect of sex on DR across both species and groups. Whether EDCs at environmentally relevant levels can significantly impact DR in these species remains an open question.

The absence of a truly uncontaminated control group compelled us to reconsider the role of amphibians as potential bioindicators of water quality, even in officially treated water sources. However, we acknowledge the methodological limitations this poses and interpret our findings with caution. This challenge highlights the broader issue of water purification efficacy in treatment plants, where conventional processes may not eliminate all ecologically relevant contaminants from the sewage and the surface runoff.

## Figures and Tables

**Table 1:** Summary of compounds, which presence in the river and control water was monitored during experiment.

Compound	Impact on Amphibians	Presence and Relevance
**Bisphenol A (BPA)**	Affected thyroid function in *Xenopus laevis*, *Xenopus tropicalis*, *Glandirana rugosa* [57,58]. Disrupted somatic development in *X. laevis*, *X. tropicalis*, *G. rugosa*, *Rhinella arenarum, Pelophylax nigromaculatus* [57,59,60,61,62]. Negatively affected testes development in *X. laevis* [63]. Feminized sex ratio in *X. laevis* [64,65,66].	One of the most widely studied endocrine-disrupting compounds (EDCs). Present in polycarbonate plastics, epoxy resins, and various consumer products. Found in surface waters, affecting wildlife and human health [6].
**Nonylphenol (NP)**	Had feminizing effect on sex ratio in *X. laevis* [64] and gonadal development in *L. pipiens*, *L. sylvaticus* [67] and *Lithobates catesbeianus* [68]. Impacted levels of sexual hormones in *Pelophylax* kl. *esculentus* [65].	A persistent environmental contaminant from the degradation of alkylphenols. Used in surfactants and detergents. Commonly found in aquatic environments, affecting reproductive functions [65].
**Parabens**	Methylparaben impaired gene expression and development in *X. laevis* embryos at relatively high concentrations [69]. Propylparaben affected the thyroid function in *X. tropicalis* [70].	Widely used as preservatives in cosmetics, pharmaceuticals, and personal care products. Frequently detected in surface waters. Emerging EDCs require further research on their effects on amphibians [71].
**Estrogens**	Estrone (E1) caused sex reversal in *Ambystoma tigrinum* [72]. 17β-estradiol (E2) had feminizing effect on sex ratio and gonadal development in *X. laevis* [66,73,74,75]; *Anaxyrus* (*Bufo) americanus, Dryophytes versicolor* and *Lithobates sphenocephalus* [76]. 17-ethinylestradiol (EE2) had a feminizing effect on sex ratio and gonadal development of *X. laevis* and *X. tropicalis* [77,78,79]; *Hyla arborea*, *Bufotes* viridis [80]; *Bufo bufo* [81], *Rana temporaria* [77], *L. pipiens* [67,82] and *L. sylvaticus* [83].	Found in surface waters and wastewater effluents, originating from natural and synthetic hormones, including those used in contraceptive pills and hormone therapies. One of the most potent EDCs affecting amphibians [84,85].
**Progesterone**	Negatively affected oogenesis and disrupted metamorphosis in *X. laevis* [86,87].	Present in the environment due to pharmaceutical use in contraceptives and cancer treatment therapies. Its effects on amphibian populations remain poorly understood, requiring further investigation [87].

**Table 2:** Concentrations of compounds in given weeks of expositions in randomly chosen control (C1-2) and experimental (E1-2) tanks with *Rana arvalis* (RA) and *Rana temporaria* (RT) tadpoles.

Compound	Week	RA C1 [µg/I]	RA C2 [µg/I]	RA E1 [µg/I]	RA E2 [µg/I]	RT C1 [µg/I]	RT C2 [µg/I]	RT E1 [µg/I]	RT E2 [µg/I]
**bisphenol A**	1	<0.095	<0.095	<0.095	<0.095	<0.095	<0.095	<0.095	<0.095
**bisphenol A**	3	<0.095	<0.095	<0.095	<0.095	<0.095	<0.095	<0.095	<0.095
**bisphenol A**	5	<0.095	<0.095	<0.095	<0.095	<0.095	<0.095	<0.095	<0.095
**bisphenol A**	7	<0.095	<0.095	<0.095	<0.095	<0.095	<0.095	<0.095	<0.095
**bisphenol A**	9	<0.095	<0.095	<0.095	<0.095	<0.095	<0.095	<0.095	<0.095
**nonylphenol**	1	N.D.	N.D.	N.D.	N.D.	0.1697	N.D.	N.D.	N.D.
**nonylphenol**	3	N.D.	N.D.	N.D.	N.D.	N.D.	N.D.	N.D.	N.D.
**nonylphenol**	5	N.D.	N.D.	N.D.	N.D.	N.D.	N.D.	N.D.	N.D.
**nonylphenol**	7	N.D.	N.D.	N.D.	N.D.	N.D.	N.D.	N.D.	N.D.
**nonylphenol**	9	N.D.	N.D.	N.D.	N.D.	N.D.	N.D.	N.D.	N.D.
**estradiol**	1	N.D.	N.D.	<0.084	N.D.	N.D.	N.D.	N.D.	<0.084
**estradiol**	3	N.D.	N.D.	N.D.	N.D.	N.D.	N.D.	N.D.	N.D.
**estradiol**	5	N.D.	N.D.	N.D.	N.D.	N.D.	N.D.	N.D.	N.D.
**estradiol**	7	N.D.	N.D.	N.D.	N.D.	N.D.	N.D.	N.D.	N.D.
**estradiol**	9	N.D.	N.D.	N.D.	N.D.	N.D.	N.D.	N.D.	N.D.
**estrone**	1	N.D.	N.D.	N.D.	<0.071	0.1640	N.D.	4.5275	<0.071
**estrone**	3	N.D.	N.D.	N.D.	N.D.	0.1100	N.D.	1.3800	N.D.
**estrone**	5	N.D.	N.D.	N.D.	N.D.	N.D.	N.D.	1.0400	N.D.
**estrone**	7	N.D.	N.D.	N.D.	N.D.	N.D.	N.D.	N.D.	N.D.
**estrone**	9	N.D.	N.D.	N.D.	N.D.	N.D.	N.D.	N.D.	N.D.
**ethinylestradiol**	1	2.0284	1.2656	2.0300	3.6314	3.4168	1.5508	5.5474	2.7144
**ethinylestradiol**	3	1.0300	0.9700	0.5500	1.4300	2.3600	1.4700	<0.098	<0.098
**ethinylestradiol**	5	<0.098	0.2200	0.3200	0.3300	<0.098	N.D.	<0.098	<0.098
**ethinylestradiol**	7	<0.098	<0.098	<0.098	<0.098	<0.098	N.D.	<0.098	0.1200
**ethinylestradiol**	9	<0.098	<0.098	<0.098	<0.098	<0.098	N.D.	<0.098	<0.098
**progesterone**	1	N.D.	N.D.	N.D.	N.D.	N.D.	N.D.	<0.098	N.D.
**progesterone**	3	N.D.	N.D.	N.D.	N.D.	N.D.	N.D.	N.D.	N.D.
**progesterone**	5	N.D.	N.D.	N.D.	N.D.	N.D.	N.D.	N.D.	N.D.
**progesterone**	7	N.D.	N.D.	N.D.	N.D.	N.D.	N.D.	N.D.	N.D.
**progesterone**	9	N.D.	N.D.	N.D.	N.D.	N.D.	N.D.	N.D.	N.D.
**methylparaben**	1	1.0400	1.1000	N.D.	N.D.	2.3227	0.8000	N.D.	N.D.
**methylparaben**	3	0.1200	0.4500	N.D.	N.D.	N.D.	0.3900	N.D.	N.D.
**methylparaben**	5	N.D.	N.D.	N.D.	N.D.	N.D.	N.D.	N.D.	N.D.
**methylparaben**	7	N.D.	N.D.	N.D.	N.D.	N.D.	N.D.	N.D.	N.D.
**methylparaben**	9	N.D.	N.D.	N.D.	N.D.	N.D.	N.D.	N.D.	N.D.
**propylparaben**	1	0.4924	0.4999	1.0016	1.0727	0.9729	0.4186	4.5851	0.7415
**propylparaben**	3	0.3900	0.2500	N.D.	0.1300	N.D.	0.2700	0.2600	0.5300
**propylparaben**	5	N.D.	N.D.	N.D.	N.D.	N.D.	N.D.	N.D.	N.D.
**propylparaben**	7	N.D.	N.D.	N.D.	N.D.	N.D.	N.D.	N.D.	N.D.
**propylparaben**	9	N.D.	N.D.	N.D.	N.D.	N.D.	N.D.	N.D.	N.D.

N.D.—not detected.

**Table 3:** Concentration of chlorophyll A and phaeophytin A in control (C1-4) and experimental (E1-4) tanks with *Rana arvalis* (RA) and *Rana temporaria* (RT) in the first and last week of exposition.

Collection Time	Water Tanks	Chlorophyll A	Phaeophytin A
		µg/I	µg/I
1st week of exposition	collective sample from all control tanks	1.28	2.08
	collective sample from all experimental tanks	17.32	3.22
9th week of exposition	RA (C1-C4) mean value (±): SD	143.29 (±): 144.81	24.58 (±): 15.95
	RT (C1-4) mean value (±): SD	33.05 (±): 29.61	28.27 (±): 18.87
	RA (E1-4) mean value (±): SD	202.88 (±): 45.52	55.61 (±): 8.89
	RT (E1-4) mean value (±): SD	257.71 (±): 146.03	73.62 (±): 28.87

SD—standard deviation.

## Data Availability

The original contributions presented in this study are included in the article/Supplementary Materials. Further inquiries can be directed to the corresponding author.

## Supplementary Materials

The following supporting information can be downloaded at https://www.mdpi.com/article/10.3390/ani15233364/s1, Table S1: Morphometric measurements: weight, snout-to-vent length (SVL), head width (HW) and Body MassIndex (BMI) in *Rana arvalis* by group and sex. Table S2: Morphometric measurements: weight, snout-to-vent length (SVL), head width (HW) and Body MassIndex (BMI) in *Rana temporaria* by group and sex. Table S3: Summary of digit ratio (2D:4D) measurements in *Rana arvalis* (RA) *and Rana temporaria* (RT), by females (F) and males (M), control (C) and experimental (E) groups, and limb (FL—fore left, FR—fore right, HL—hind left, HR—hind right). Figure S1: Mean temperature, pH, and conductivity in subsequent weeks of exposition in the control (C) and experimental (E) group. Figure S2: Comparison of temperature, pH and conductivity across tanks in the control (C) and experimental (E) groups. Figure S3: SVL distribution by species, group, and sex. Figure S4: Weight distribution by species, group, and sex. Figure S5: Relationship between snout–vent length (SVL) and adjusted digit ratio (DR_adj) in left (HL) and right (HR) hind limbs in females (F) and males (M) in (a,b): *Rana arvalis*, the experimental group; (c,d): *R. arvalis*, the control group; (e,f): *Rana temporaria*, the experimental group; (g,h): *R. temporaria*, the control group. A significant positive correlation was observed between SVL and DR_adj, indicating that larger individuals had higher 2D:4D digit ratios.
