# Supplementary material for: Investigating Sexual Characteristics in Two Frog Species Under Exposure to River Water Polluted with Endocrine Disruptors
Source: Animals (Basel). 2025 Nov 21;15(23):3364. doi: 10.3390/ani15233364 (PMC12691299; doi:10.3390/ani15233364)
Supplement: Supplementary file 1 [file animals-15-03364-s001.zip › Table S1 Rana temporaria variables.pdf]

Table S1. Morphometric measurements: weight, snout-to-vent length (SVL), head width (HW) and Body Condition Index (BMI) in *Rana arvalis* by group and sex.

Group	sex	Variable	n	min	max	mean	SD	SE
**control**	females	weight	39	0,36	86	3,353	13,591	2,176
		HW	39	4,62	21,82	7,069	3,207	0,514
		SVL	39	6,37	27,72	21,785	3,454	0,553
		BMI	39	0,022	3,941	0,157	0,623	0,1
	males	weight	52	0,43	2,66	1,129	0,497	0,069
		HW	52	4,99	24,96	7,19	3,937	0,546
		SVL	52	7,76	27,67	21,734	3,27	0,453
		BMI	52	0,024	0,343	0,054	0,043	0,006
**experimental**	females	weight	44	0,57	1,92	1,16	0,296	0,045
		HW	44	5,35	7,14	6,343	0,435	0,066
		SVL	44	17,45	26,9	22,386	1,883	0,284
		BMI	44	0,032	0,074	0,051	0,01	0,001
	males	weight	46	0,61	2,4	1,341	0,449	0,066
		HW	46	5,59	7,82	6,596	0,526	0,078
		SVL	46	18,9	28,67	23,491	2,363	0,348
		BMI	46	0,031	0,09	0,056	0,014	0,002
