# Supplementary material for: Investigating Sexual Characteristics in Two Frog Species Under Exposure to River Water Polluted with Endocrine Disruptors
Source: Animals (Basel). 2025 Nov 21;15(23):3364. doi: 10.3390/ani15233364 (PMC12691299; doi:10.3390/ani15233364)
Supplement: Supplementary file 1 [file animals-15-03364-s001.zip › Table S2.pdf]

Table S2. Morphometric measurements: weight, snout-to-vent length (SVL), head width (HW) and Body Condition Index (BMI) in *Rana temporaria* by group and sex.

group	sex	variable	n	min	max	mean	sd	se
**control**	females	weight	6	0,86	1,39	1,135	0,196	0,08
		HW	6	5,72	6,76	6,363	0,386	0,158
		SVL	6	21,92	26,7	23,603	1,669	0,681
		BMI	6	0,039	0,055	0,048	0,006	0,002
	males	weight	7	0,79	2,61	1,687	0,7	0,264
		HW	7	6,69	7,89	7,377	0,543	0,205
		SVL	7	21,87	31,75	26,287	3,671	1,388
		BMI	7	0,036	0,086	0,062	0,018	0,007
**experimental**	females	weight	15	0,67	1,43	0,987	0,232	0,06
		HW	15	5,7	6,96	6,283	0,37	0,095
		SVL	15	19,77	25,21	22,225	1,384	0,357
		BMI	15	0,032	0,057	0,044	0,008	0,002
	males	weight	12	0,79	2,7	1,323	0,517	0,149
		HW	12	5,66	7,78	6,646	0,534	0,154
		SVL	12	20,84	30,61	23,892	2,647	0,764
		BMI	12	0,037	0,088	0,054	0,014	0,004
