# Supplementary material for: Investigating Sexual Characteristics in Two Frog Species Under Exposure to River Water Polluted with Endocrine Disruptors
Source: Animals (Basel). 2025 Nov 21;15(23):3364. doi: 10.3390/ani15233364 (PMC12691299; doi:10.3390/ani15233364)
Supplement: Supplementary file 1 [file animals-15-03364-s001.zip › Table S3 DR stats.pdf]

Table S3. Summary of digit ratio (2D:4D) measurements in *Rana arvalis* (RA) and *Rana temporaria* (RT), by females (F) and males (M), control (C) and experimental (E) group, and limb (FL – fore left, FR - fore right, HL – hind left, HR – hind right) with descriptive statistics (SD – standard deviation, SE – standard error).

Species	Sex	Group	Limb	Min	Max	Mean	SD	SE
RA	F	C	FL	0,618	0,952	0,781	0,061	0,010
			FR	0,583	0,924	0,781	0,071	0,011
			HL	0,288	0,380	0,341	0,021	0,003
			HR	0,296	0,378	0,334	0,020	0,003
		E	FL	0,596	0,911	0,756	0,077	0,012
			FR	0,556	0,858	0,774	0,051	0,008
			HL	0,280	0,380	0,322	0,026	0,004
			HR	0,282	0,378	0,327	0,023	0,003
	M	C	FL	0,581	1,002	0,742	0,081	0,011
			FR	0,647	1,206	0,762	0,082	0,011
			HL	0,255	0,384	0,335	0,023	0,003
			HR	0,243	0,468	0,332	0,034	0,005
		E	FL	0,588	0,902	0,766	0,057	0,008
			FR	0,679	0,936	0,783	0,050	0,007
			HL	0,283	0,398	0,317	0,023	0,003
			HR	0,269	0,373	0,324	0,022	0,003
RT	F	C	FL	0,494	0,827	0,677	0,109	0,036
			FR	0,490	0,841	0,690	0,118	0,039
			HL	0,290	0,355	0,338	0,022	0,008
			HR	0,319	0,382	0,350	0,023	0,009
		E	FL	0,643	0,869	0,750	0,061	0,016
			FR	0,689	0,845	0,766	0,041	0,010
			HL	0,297	0,424	0,350	0,035	0,009
			HR	0,293	0,387	0,346	0,027	0,007
	M	C	FL	0,554	0,810	0,714	0,078	0,026
			FR	0,490	0,824	0,671	0,129	0,039
			HL	0,326	0,360	0,344	0,011	0,004
			HR	0,301	0,380	0,340	0,026	0,010
		E	FL	0,582	0,781	0,701	0,056	0,016
			FR	0,666	1,153	0,756	0,128	0,037
			HL	0,288	0,421	0,344	0,038	0,011
			HR	0,301	0,362	0,334	0,019	0,006
