# Supplementary figures and images for: Investigating Sexual Characteristics in Two Frog Species Under Exposure to River Water Polluted with Endocrine Disruptors
Source: Animals (Basel). 2025 Nov 21;15(23):3364. doi: 10.3390/ani15233364 (PMC12691299; doi:10.3390/ani15233364)

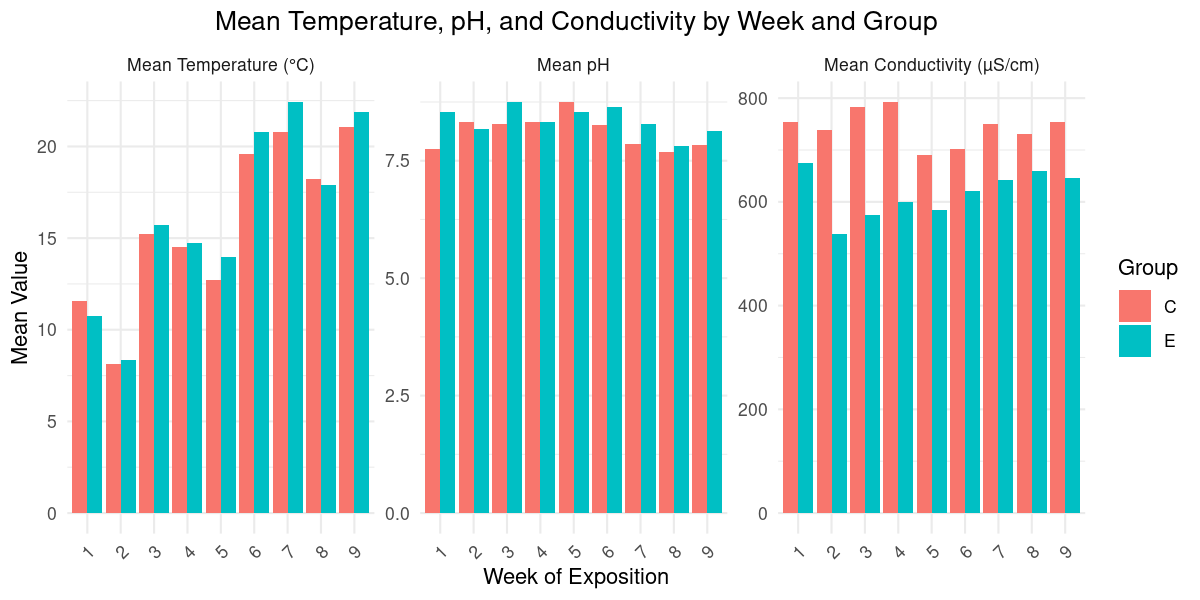

Supplement: Supplementary file 1 [file animals-15-03364-s001.zip › Figure S1 Mean temperature pH conductivity weeks and groups.png]

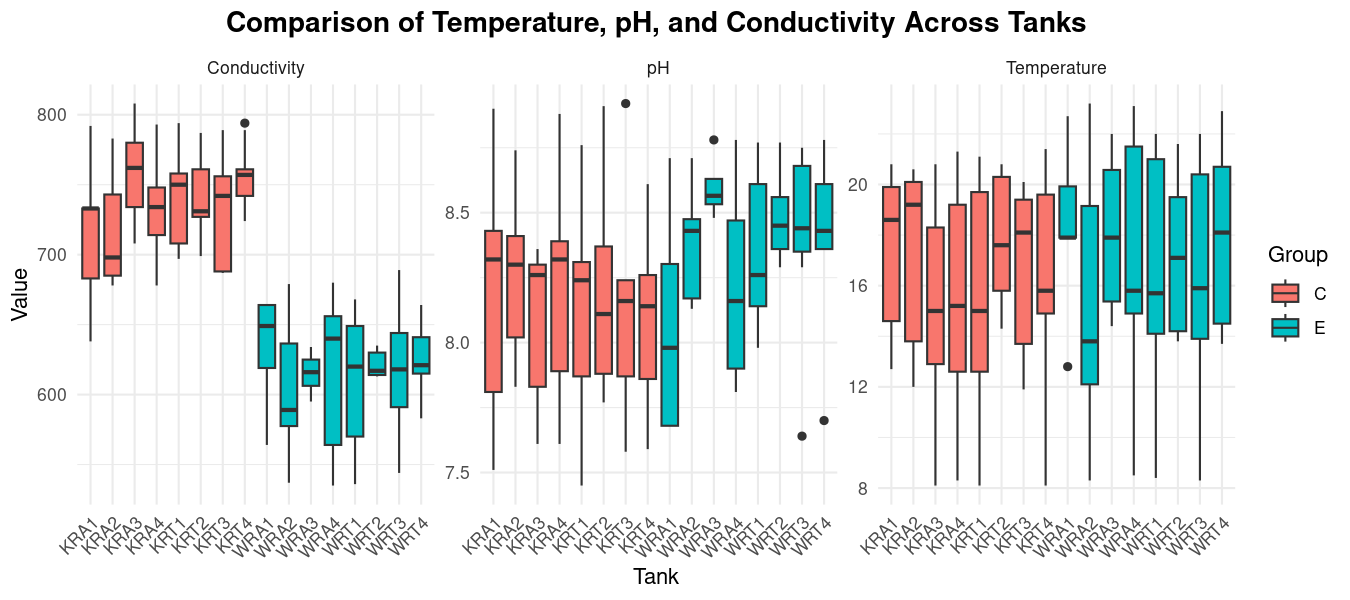

Supplement: Supplementary file 1 [file animals-15-03364-s001.zip › Figure S2 Comparison of T pH Cond across tanks.png]

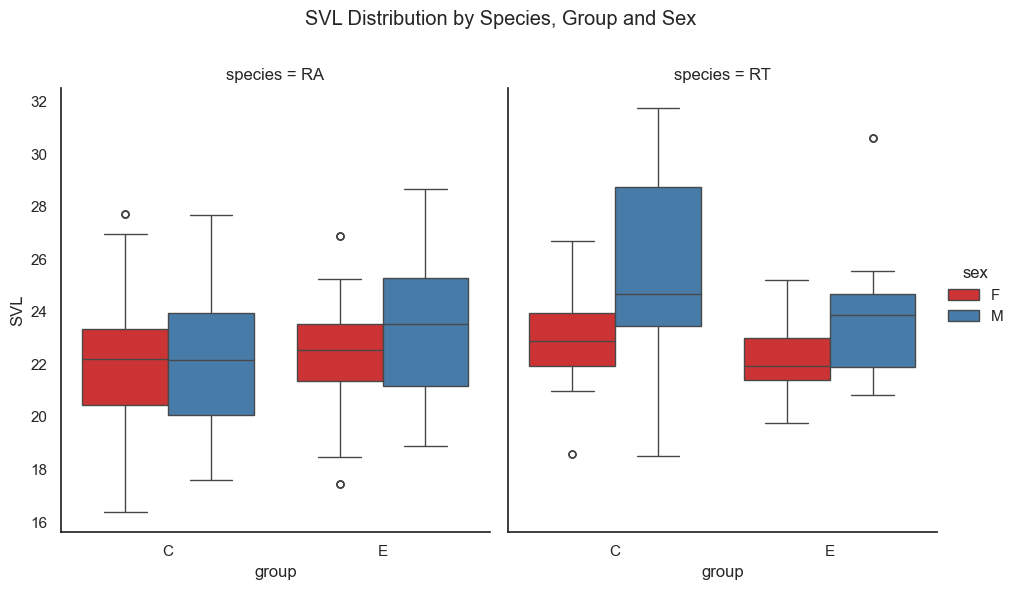

Supplement: Supplementary file 1 [file animals-15-03364-s001.zip › Figure S3 Distr SVL.png]

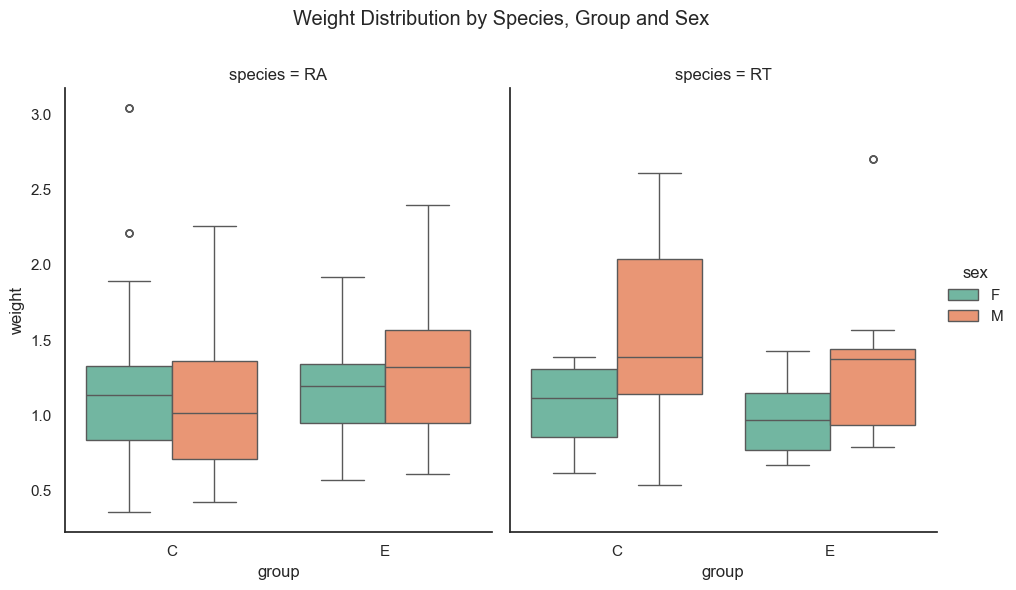

Supplement: Supplementary file 1 [file animals-15-03364-s001.zip › Figure S4 Distr weight.png]

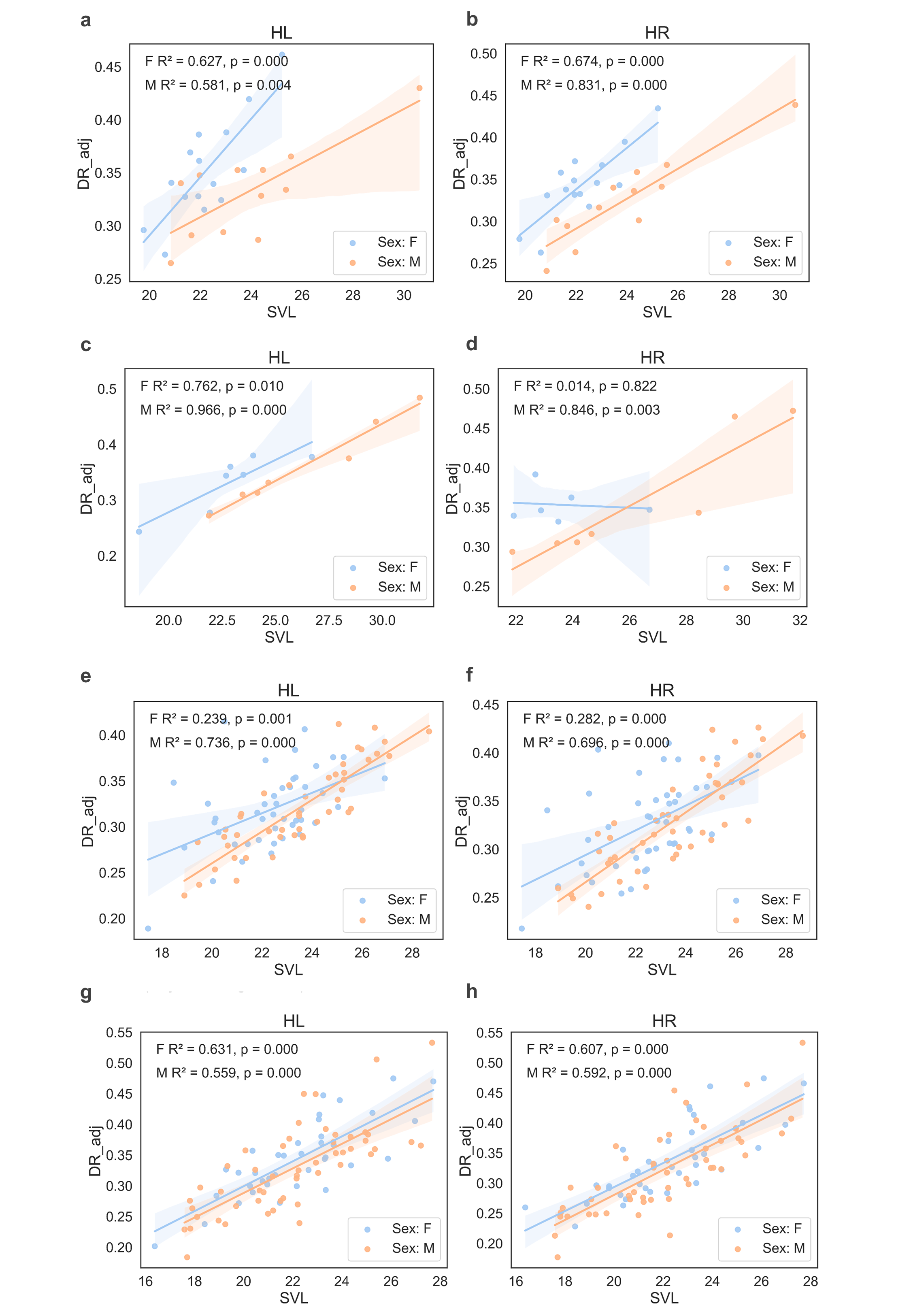

Supplement: Supplementary file 1 [file animals-15-03364-s001.zip › Figure S5 SVL DR regression hindlimbs.png]
